# Enhanced Photocatalytic Wastewater Purification and Bacterial Elimination via Copper Ion Irradiation‐Induced Modification of BiOCl

**DOI:** 10.1002/advs.202523633

**Published:** 2026-01-22

**Authors:** Sihan Ma, Dewang Cui, Jianglong Kong, Wentao Li, Zheng Han, Yipeng Li, Shidong Zhang, Xinglin Yu, Deng Long, Xue Bai, Lin Wang, Guang Ran, Zhijun Zhao

**Affiliations:** ^1^ College of Big Data and Information Engineering Guizhou University Guiyang China; ^2^ College of Energy Xiamen University Xiamen China; ^3^ Department of Food Nutrition and Safety/National R&D Center Herbal Medicine, Processing, College of Engineering China Pharmaceutical University Nanjing China; ^4^ Qujing University of Medicine & Health Sciences Qujing China; ^5^ School of Physics Beihang University Beijing China; ^6^ School of Biomedical Engineering Capital Medical University Beijing China; ^7^ Department of Oncology, School of Medicine, Zhongshan Hospital of Xiamen University Xiamen University Xiamen China; ^8^ Central Laboratory Peking University First Hospital Ningxia Women and Children's Hospital (Ningxia Hui Autonomous Region Maternal and Child Health Hospital) Yinchuan China; ^9^ Third Clinical Medical College Ningxia Medical University Yinchuan China

**Keywords:** copper ion implantation, disinfection, modification engineering, photocatalytic degradation, surface electric potential gradient

## Abstract

The BiOCl photocatalyst possesses a relatively weak inherent intrinsic electric field property, which significantly hampers the separation efficiency of photogenerated carriers and, consequently, limits the full exploitation of its application potential. Reliably addressing this issue stands as an outstanding challenge in the quest to improve the catalyst's activity. In this study, we utilized carrying‐energy copper ion beam irradiation to modify BiOCl, aiming to controllably bolster its intrinsic electric field. Through meticulous adjustment of the copper ion beam irradiation dosages, we achieved controlled local structural reconstructions in BiOCl. The reconstructions notably altered the surface potential gradient, thereby amplifying the efficacy of the intrinsic built‐in electric field, inhibiting the recombination of photogenerated carriers, and ultimately leading to remarkable improvements in photocatalytic performance. As a result, the irradiated‐BiOCl composite demonstrated a 3.5‐fold in RhB degradation rate compared to pristine BiOCl under light irradiation, accompanied by complete inactivation of *E. coli* within 15 min. The inspiration of copper ion beam irradiation engineering presents a scalable approach for advancing multifunctional photocatalysis in environmental remediation and public health applications.

## Graphical abstract

This study presents a copper ion irradiation method to achieve the modification function of BiOCl. By using high‐energy ion beams to introduce impurity ions inside the material, the intrinsic electric field of BiOCl is optimized, promoting the separation of photogenerated carriers and enhancing the photocatalytic activity. Ultimately, high‐performance photocatalytic water treatment and bacterial removal are achieved.

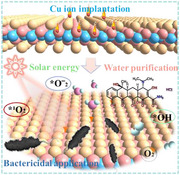

## 1 Introduction

The rapid surge in the global population, coupled with the swift advancement of industrialization, has triggered severe environmental challenges [1, 2, 3]. These are marked by the widespread generation and proliferation of hazardous waste, harmful bacteria, and organic pollutants [4, 5, 6]. Organic compounds [7], encompassing dyes [8, 9, 10] and pharmaceutical by‐products [11, 12, 13, 14, 15], continuously seep into water systems, causing extensive pollution and posing a threat not only to the sustainable health of humanity but also to the long‐term safety and stability of aquatic ecosystems [16]. More than 600 million people living in rural areas of low‐income and middle‐income countries still do not have access to basic drinking water services [17]. Relevant reports indicate that a staggering 4.5 billion people lack access to safe drinking water and proper sanitation facilities, with over 500,000 annual deaths attributed to the consumption of contaminated water [18]. Therefore, there exists an urgent imperative to explore and implement effective technological solutions for addressing water pollutants, thereby safeguarding the stable and sustainable progression of society. Advanced oxidation processes (AOPs) are widely recognized as one of the most promising treatment approaches for the elimination of stubborn organic pollutants from wastewater [19, 20]. Through the production of highly reactive oxygen species (ROS) with potent oxidizing and reducing capabilities, such as hydroxyl radicals (•OH), singlet oxygen (^1^O_2_), superoxide anions (•O_2_^−^), and hydrogen peroxide (H_2_O_2_), facilitates the thorough mineralization of organic contaminants [21] and the highly efficient removal of harmful bacteria [22]. For example, Zhao et al. employed light‐activated Bi/BiOI to produce high‐intensity ROS, effectively eliminating harmful bacteria [23]. Similarly, Ge et al. utilized Cu_1_O_x_ to specifically catalyze hydrogen peroxide, generating highly reactive ROS that achieved excellent performance in both pollutant degradation and sterilization [24]. These outstanding contributions strongly demonstrated the effectiveness and environmental friendliness of AOPs in the water treatment process. Among the array of AOPs available, photocatalysis has emerged as a focal point of considerable interest, largely due to its extraordinary proficiency in decomposing organic pollutants and removing harmful bacteria without relying on biological or chemical treatments. Combined with its economic viability and environmentally benign characteristics, renders it a highly appealing wastewater solution [25].

BiOCl photocatalyst has garnered substantial attention owing to its intriguing layered structural attributes, and the periodic arrangement of [Bi_2_O_2_]^2+^ and [Cl_2_]^2^^−^ layers facilitated by van der Waals forces [26, 27, 28]. This unique configuration generates a beneficial internal electric field (IEF), enhancing its photocatalytic performance. However, the inherent built‐in electric field remains relatively weak, making it inadequate to fully suppress the recombination of photogenerated carriers. As a result, this limits the effective attainment of high‐performance photocatalysis. Therefore, by modulating the intrinsic electric field strength, it is feasible to enhance the separation and migration efficiency of photogenerated carriers, thereby improving carrier utilization. However, optimizing controllably the intrinsic electric field's effectiveness for targeted enhancement of photocatalytic performance continues to pose a substantial challenge.

Recently, ion irradiation technology has emerged as a highly promising material modification technique, capable of precisely tailoring the physical and chemical properties of materials [29, 30, 31]. Unlike chemical modification approaches, physical methods such as ion irradiation primarily induce structural alterations in materials through ion‐lattice interactions, thereby triggering concurrent physical and chemical transformations [32, 33, 34]. Interestingly, ion irradiation offers distinct advantages, including localized precision control and tunable energy/dose regulation, making it highly promising for targeted material property modification [35, 36, 37]. Impressively, in catalytic performance engineering, it enables the introduction of active sites [38], controlled doping and defects [39, 40, 41, 42], and heterostructure construction [43, 44], morphological [45] and size regulation [46], all while enhancing catalytic activity without secondary pollutants. This approach combines convenience, speed, cost‐effectiveness, and environmental sustainability.

Precious metals like gold and silver are renowned for their ability to exhibit surface plasmon resonance (SPR), a phenomenon that significantly amplifies catalytic activity by generating intense localized electromagnetic fields near the metal surface. However, copper, which is far more abundant and cost‐effective, also demonstrates comparable plasmon resonance capabilities. Notably, in catalytic processes, copper not only achieves this effect but also showcases exceptional performance, making it a promising alternative to noble metals. Herein, we ingeniously employed Cu ion implantation technology to locally reconfigure the structural characteristics of the BiOCl photocatalyst by precisely controlling the irradiation dose. This approach aimed to amplify catalytic activity by promoting the generation of photogenerated carriers through an enhanced intrinsic electric field. Notably, varying ion implantation doses lead to distinct ion densities, which induce significant local structural modifications in BiOCl and enable precise control over doping concentration. Consequently, this results in a controllable gradient change in the surface electric potential of BiOCl, facilitating effective modulation of the intrinsic electric field. The results demonstrate that BiOCl photocatalysts subjected to ion implantation exhibit significantly enhanced photocatalytic degradation capabilities, with a 250% increase in degradation efficiency. Furthermore, they can achieve complete inactivation of *E. coli* within 15 min under low‐intensity light illumination. These findings indicate that ion implantation engineering is a controllable and effective strategy for modulating catalytic activity, offering a promising methodological approach for the precise design of high‐performance catalysts.

## 2 Results and Discussion

### 2.1 Synthesis and Characterization of BiOCl and BiOCl‐irrX

**FIGURE 1:**
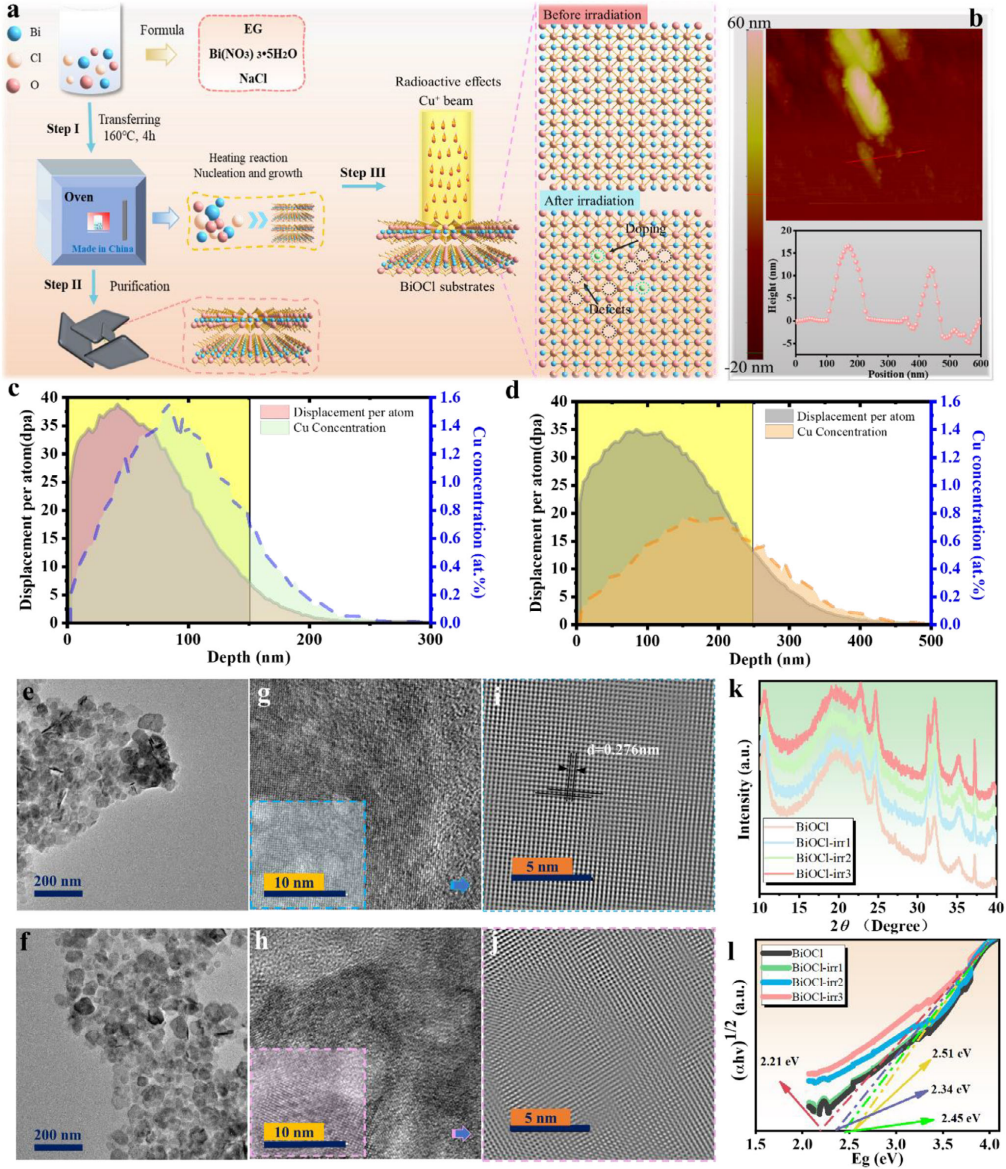
(a) Schematic illustration of BiOCl and copper ion implantation engineering. (b) AFM image of BiOCl. (c) SRIM calculation for 200 kV copper ion irradiated BiOCl with electronic and nuclear energy stopping (Irradiation dose: 10^16^ ions cm^−2^). (d) SRIM calculation for 400 kV copper ion irradiated BiOCl with electronic and nuclear energy stopping (Irradiation dose: 10^16^ ions cm^−2^). TEM images of BiOCl before (e) and after irradiation (f). High‐resolution TEM images of BiOCl before (g) and after irradiation (h). Inverse fast Fourier transform image of BiOCl before (i) and after irradiation (j). (k) XRD pattern of BiOCl. (l) Ultraviolet‐visible diffuse reflection spectra (DRS) of BiOCl.

We employed a solvothermal process to synthesize the BiOCl product, and subsequently subjected it to modification by irradiating it with a copper ion beam generated using an ion accelerator (Figure 1a). During the ion irradiation process, an ion acceleration energy of 400 kV was deliberately chosen by the designed multiple‐ion beam accelerator and in‐situ TEM system (Figures S1 and S2). Subsequently, various irradiation doses, namely 5 × 10^12^, 1 × 10^13^, and 5 × 10^13^ ions cm^−2^, can be precisely controlled and adjusted. Following the established preparation protocol for BiOCl, we successfully synthesized nanosheets exhibiting a notably uniform morphology. Utilizing atomic force microscopy (AFM), we precisely determined that the thickness of these nanosheets hovered around 15 nm (Figure 1b).

Leveraging the unique interaction dynamics between copper ions and BiOCl, the irradiation parameters were meticulously evaluated and calculated through the utilization of the Simulation of Radiation Induced Materials Effects (SRIM) software [47, 48, 49], thereby guaranteeing the reliable execution of the experimental procedure. As shown in Figure 1c,d, the simulations of ion irradiation were meticulously carried out, employing copper ions with distinct energy levels of 200 and 400 kV, respectively. The cumulative ion dosage for the irradiation process was set at 10^16^ ions cm^−2^. The outcomes revealed a discernible trend where the depth of ion penetration escalated in tandem with the augmentation of energy. Furthermore, variations in the irradiation doses exerted a discernible influence on the concentration of ion implantation, consequently affecting the precision with which the ion doping concentration could be controlled.

The morphological features of BiOCl subjected to various treatment methods are vividly depicted in Figure 1e,f. The findings reveal that both the initially prepared BiOCl and the BiOCl post‐irradiation treatment maintain a distinctive two‐dimensional sheet‐like architecture. Further scrutiny into the atomic lattice arrangement was undertaken via high‐resolution transmission electron microscopy (HRTEM), with the resultant data presented in Figure 1g,h. Notably, an observable uptick in the quantity of local atomic defects becomes apparent. By employing inverse fast Fourier transform (IFFT) techniques, dislocation structures within the local atomic lattice configuration are more distinctly discernible (Figure 1i,j). This phenomenon can be attributed to the introduction of lattice atomic defects in BiOCl, precipitated by the copper ion implantation process, along with lattice deformation stemming from localized doping effects during the copper ion implantation (Figure S3) [50, 51, 52]. SEM analysis revealed a pronounced trend: as the irradiation dose escalated, its impact on BiOCl intensified notably. The surface morphology exhibited a tendency to become increasingly rugged, with the boundaries between distinct shapes becoming progressively indistinct. This observation substantiates the significant influence exerted by the ion irradiation modification process on the surface morphology of BiOCl [53]. The emergence of ion beam irradiation effects exerts a profound influence on the material's microstructure, subsequently impacting its physical and chemical attributes. XRD analysis provided insightful revelations into the crystal structural attributes of BiOCl throughout various irradiation stages (Figure 1k). Notably, as the ion irradiation dose intensified, BiOCl‐irr3 displayed a subtle rightward shift in its characteristic peak. This phenomenon can be ascribed to the deposition of implanted copper ions within the BiOCl atomic lattice, coupled with the formation of a greater abundance of defect sites. Furthermore, to ascertain the structural transformations in BiOCl following ion beam irradiation modification, with the overarching goal of optimizing its photocatalytic efficacy and augmenting light absorption capabilities, we undertook diffuse reflection measurements. The outcomes of these tests are visually represented in the Figure 1l. The light absorption capacity of BiOCl exhibits a gradual enhancement post‐irradiation (Figure S5). Utilizing Lambert‐Beer's law, we were able to ascertain the corresponding band gap widths: the pristine BiOCl possesses a band gap of 2.51 eV, whereas the irradiated variants BiOCl‐irr1, BiOCl‐irr2, and BiOCl‐irr3 display band gaps of 2.45, 2.34, and 2.21 eV, respectively. This outcome underscores that the precisely controlled infusion of copper ions enables the modulation of the band gap, effectively broadening the light absorption spectrum to encompass a more advantageous range.

**FIGURE 2:**
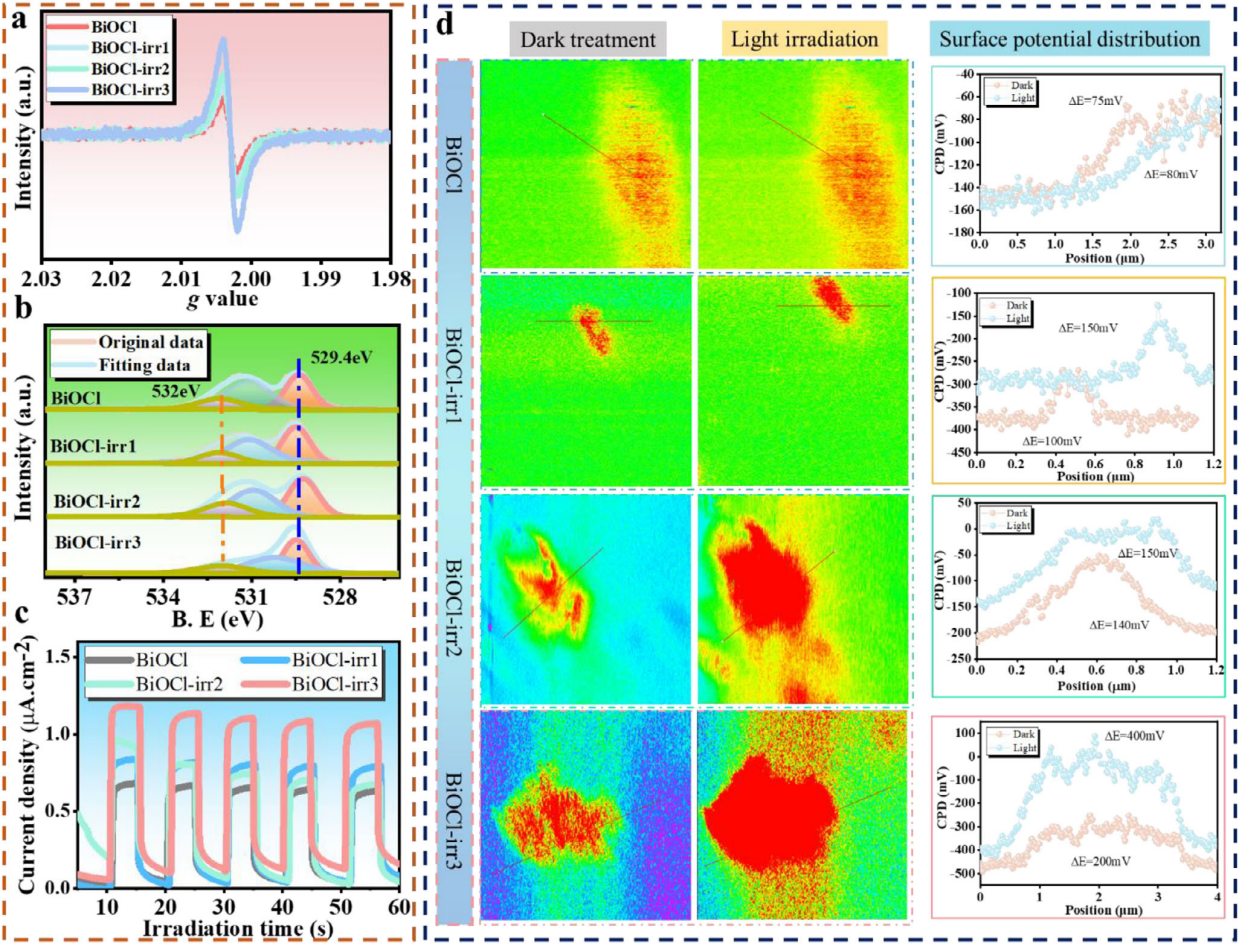
(a) The EPR spectra of BiOCl treated with/without Cu ion irradiation. (b) XPS refine spectra of O*1s*. (c) The transient photocurrent response curves of various samples. (d) The surface potentials of various BiOCl samples.

The interaction between ions and the atoms within the material triggers atomic displacement as energy is transferred. Specifically, when the energy imparted surpasses the threshold necessary for atomic movement, vacancies emerge as a form of defect. Utilizing electron paramagnetic resonance (EPR) spectroscopy, we can directly glean insights into these vacancy defects. The observation of a g‐value equal to 2.003 provides compelling evidence that an escalation in the irradiation dose correlates with a heightened quantity of oxygen vacancy defects within the BiOCl lattice (Figure 2a). This discovery is also consistent with the result observed by HRTEM, which indicated an increase in defects. Through the application of X‐ray photoelectron spectroscopy (XPS) to scrutinize the bonding characteristics and chemical composition of the materials, as illustrated in Figure 2b, we discerned that the Bi─O bond energy in BiOCl registers at 530.8 eV, the bond energy for adsorbed oxygen stands at 532 eV, and the Cl─O bond energy is measured at 529.4 eV [25, 54, 55]. Following Cu ion irradiation, the positions of various characteristic peaks exhibited a shift towards lower binding energies. The Bi *4f* fine spectrum analysis further revealed that the characteristic peaks migrated towards higher binding energies post‐irradiation, signifying that the irradiation process exerted an influence on the local electronic dynamics (Figure S6). In other words, the shift in peak position could be ascribed to a modification in the local electronic characteristics of the material itself. Specifically, the outer electrons of the Bi atoms migrate towards the O atoms, thereby enhancing the local electron density around the O atoms [27, 56], further enhancing electron utilization and culminating in superior catalytic activity. XPS analysis of the Cl *2p* spectra reveals a discernible leftward shift in peak positioning (Figure S7), aligning harmoniously with the observed leftward displacement in the Cl─O peak outcomes. This shift is intricately linked to a concurrent local charge transfer phenomenon. Varying irradiation dosages engender distinct Cu ion densities within the material. To attain optimal catalytic performance, a deeper exploration into the charge separation dynamics of these BiOCl nanosheets was conducted via transient photocurrent measurements, as depicted in Figure 2c. In stark contrast to the pristine BiOCl nanosheets, the BiOCl‐irr3 nanosheets manifested a notably elevated photocurrent intensity, thereby underscoring their enhanced charge separation kinetics. Furthermore, the carrier separation effect was visually confirmed through the utilization of a Kelvin probe force microscope (KPFM) [57], with the findings presented in Figure 2d. In comparison to the original BiOCl, the irradiated BiOCl samples exhibited a pronounced gradient enhancement in the surface potential difference following illumination. This outcome suggests that the process of copper ion implantation has induced structural modifications in the catalyst, leading to the formation of a more robust intrinsic built‐in electric field within the irradiated BiOCl specimens. Such an enhancement is instrumental in promoting the efficient separation and transfer of photo‐induced carriers [58, 59]. All these compelling findings unequivocally demonstrate that BiOCl, modified through copper ion implantation, exhibits enhanced light absorption capabilities and an improved intrinsic built‐in electric field, thereby establishing a solid methodological foundation for the controlled augmentation of photocatalytic activity.

### 2.2 Photocatalytic Degradation

**FIGURE 3:**
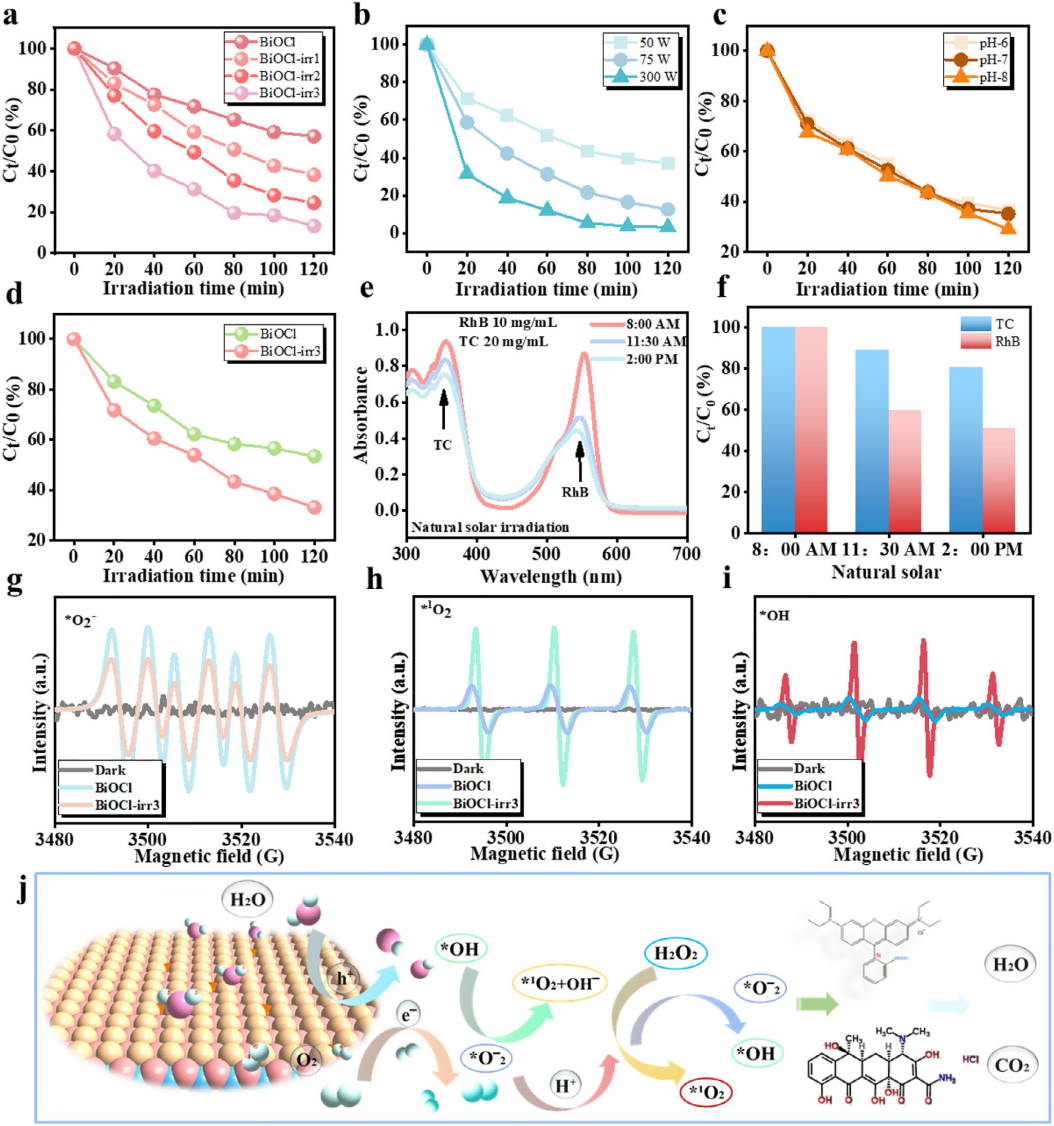
(a) Photocatalytic degradation curves of BiOCl and BiOCl‐irr3 in RhB solution. (b) The degradation comparisons of BiOCl‐irr3 in various light irradiation powers. (c) Photocatalytic degradation curves of various pH values. (d) The degradation analysis of TCH solution. (e) Changes in UV‐vis absorption spectra in TCH/RhB mixed solution under natural light photocatalysis. (f) The degradation rates of TCH and RhB under sunlight. (g) Oxygen free radical comparison analysis. (h) The comparison analysis of singlet oxygen. (i) Detection of hydroxyl radicals. (j) Schematic illustration of possible pathways of free radical generation.

Photocatalysis is widely acknowledged as a highly effective approach for treating pollutants. In this study, common pollutants, including Rhodamine B (RhB) and Tetracycline hydrochloride (TCH), were selected as research subjects to explore the alterations in the catalytic activity of the BiOCl photocatalyst following ion irradiation modification. Drawing upon prior characterization and test analyses, BiOCl‐irr3 demonstrates outstanding light absorption and carrier separation abilities. In assessing the photocatalytic performance of RhB, the degradation efficiency of the photocatalytic process is evaluated by analyzing the characteristic peak associated with RhB (Figure S8). As shown in Figure 3a, the photocatalytic degradation curve for RhB reveals that BiOCl‐irr3 exhibits superior photocatalytic degradation performance, substantiating that the precise introduction of copper ions to induce structural modifications in BiOCl significantly boosts its photocatalytic activity. Figure S9 illustrates the degradation kinetics of the photocatalysts BiOCl (k = 0.00482 min^−1^) and BiOCl‐irr3 (k = 0.01618 min^−1^), revealing that the degradation rate constant of BiOCl‐irr3 is ∼3.5 times higher than that of BiOCl, thereby underscoring its superior performance. Subsequently, the additional evidence further corroborates the reusability of BiOCl‐irr3. The findings indicate a notably consistent degradation efficiency, with roughly 80% of the degradation rate preserved even after undergoing four successive cycles of photocatalysis (Figure S10). Research investigating the degradation of RhB was carried out across diverse water environments, with the findings presented in Figures S11 and S12. In the tap water scenario, a discernible reduction in degradation efficiency and degradation kinetic constant are noted; however, this did not significantly compromise the overall degradation outcome, indicating the available degradation process. This phenomenon is likely attributable to the interference caused by an abundance of impurity ions and other variables present in tap water, which may hinder the catalytic process. Figure 3b illustrates the degradation efficiency of RhB under varying light intensities, revealing that BiOCl‐irr3 demonstrates photocatalytic activity that is contingent upon light power. The kinetics of the corresponding degradation reactions corroborate the influence of enhanced light power on catalytic activity; however, this effect does not exhibit a linear increase (Figure S13). Taking into account the potential impact of acidic or alkaline conditions within sewage systems on catalytic activity, Figure 3c indicates that the catalytic degradation performance remains robust in both mildly acidic and mildly alkaline environments. In comparison to some notable research endeavors focused on the photocatalytic degradation of RhB (Figure S14), the current study has achieved significant advancements in enhancing catalytic activity through the precise and controlled introduction of ions.

Antibiotic medications have emerged as a novel category of organic pollutants. Among these, TCH stands out as a widely utilized broad‐spectrum antibiotic, employed for treating infections in both humans and animals [60]. However, its excessive release into aquatic environments not only fosters bacterial resistance but also escalates toxicity levels for all organisms within the aquatic ecosystem. Consequently, the ion irradiated engineered BiOCl photocatalyst was employed to delve deeper into the photocatalytic degradation process of TCH. Quantitative analysis of the degradation efficiency was conducted by pinpointing the distinctive peaks within the UV‐vis absorption spectrum obtained during the photocatalytic degradation process of TCH (Figure S15). Figure 3d presents a comparative analysis of the photocatalytic degradation effects of BiOCl and BiOCl‐irr3 on TCH post‐settlement. In contrast to the pristine BiOCl, BiOCl‐irr3 demonstrates a superior degradation capability, attaining a 66.9% degradation efficiency following a 120 min illumination period, whereas BiOCl achieves merely a 46.6% degradation efficiency under identical conditions. The reaction kinetics constant reveals that BiOCl‐irr3 exhibits a superior degradation efficiency compared to BiOCl, with its catalytic activity having been augmented by roughly 70% (Figure S16). Following four consecutive cycles of testing, the degradation efficiency of TCH by BiOCl‐irr3 persisted at around 60%, underscoring its notable stability in the degradation process of TCH (Figure S17). Likewise, within the tap water system, the application of BiOCl‐irr3 exerts a negligible influence on the degradation efficiency of TCH (Figures S18 and S19), indicating an effective degradation process. The catalyst's light power‐dependent characteristics also apply to the degradation process of TCH. Elevating the light power not only boosts the degradation rate but also augments the reaction kinetics constant (Figures S20 and S21). Figure S22 provides a compelling comparison of the kinetic constants associated with TCH degradation across various catalysts, indicating that BiOCl‐irr3 exhibits a notably advantageous degradation kinetics process, despite presenting certain distinctions from the most prominent studies. Moreover, to more accurately emulate the degradation dynamics of mixed pollutants in contaminated water under real‐world scenarios, we conducted a photocatalytic degradation study of a mixture comprising RhB and TCH, utilizing natural light as the illumination source. As shown in Figure 3e, the light absorption characteristic curve of the mixture exhibits notable alterations over time when subjected to light exposure, with both the characteristic peaks of TCH and RhB displaying a discernible downward trajectory. It was distinctly evident that the degradation rate of RhB surpassed that of TCH, substantiating the notion that RhB is more susceptible to degradation than TCH, further demonstrates the competitive degradation advantage of catalytic activity (Figure 3f). Consequently, when employing an identical catalyst dosage, the reaction rate constant for RhB exceeds that of TCH, aligning harmoniously with previously documented findings. Additionally, the presence of ROS components during the photocatalytic process was confirmed through Electron Paramagnetic Resonance (EPR) analysis, with the findings depicted in Figure 3g–i. In comparison to pristine BiOCl, BiOCl‐irr3 exhibits an elevated utilization efficiency of free radicals. The ROS generated by BiOCl‐irr3 encompass not only •O_2_^−^ but also feature heightened concentrations of ^1^O_2_ and •OH when juxtaposed with the original BiOCl. This observation serves to further substantiate that BiOCl‐irr3 possesses a superior photocatalytic activity relative to its unmodified counterpart, and the satisfactory separation efficiency of photogenerated carriers induced by BiOCl‐irr3 (Figures S23 and S24). While BiOCl‐irr3 demonstrates enhanced carrier utilization and the capacity to generate a greater abundance of free radicals, it produces comparatively fewer •O_2_^−^. This discrepancy could potentially stem from the interconversion among various free radical species, as illustrated in Figure 3j. The O_2_ reacts with protons present in water, consumption of electrons, leading to the formation of ^1^O_2_ and H_2_O_2_. Subsequently, H_2_O_2_ undergoes a reaction with •O_2_^−^, yielding •OH and ^1^O_2_. Consequently, the reaction system experiences an elevation in the levels of •OH and ^1^O_2_, which in turn causes a reduction in the concentration of anions. Drawing on a wealth of prior studies, the transformation dynamics of free radicals can be succinctly outlined as follows [12, 61, 62].

$$O_{2}+e^{-}\to \cdot O_{2}^{-}$$

$$H_{2}O+h^{+}\to \cdot OH+H^{+}$$

$$O_{2}+2e^{-}+2H^{+}\to H_{2}O_{2}$$

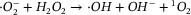




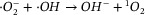




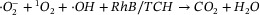




**FIGURE 4:**
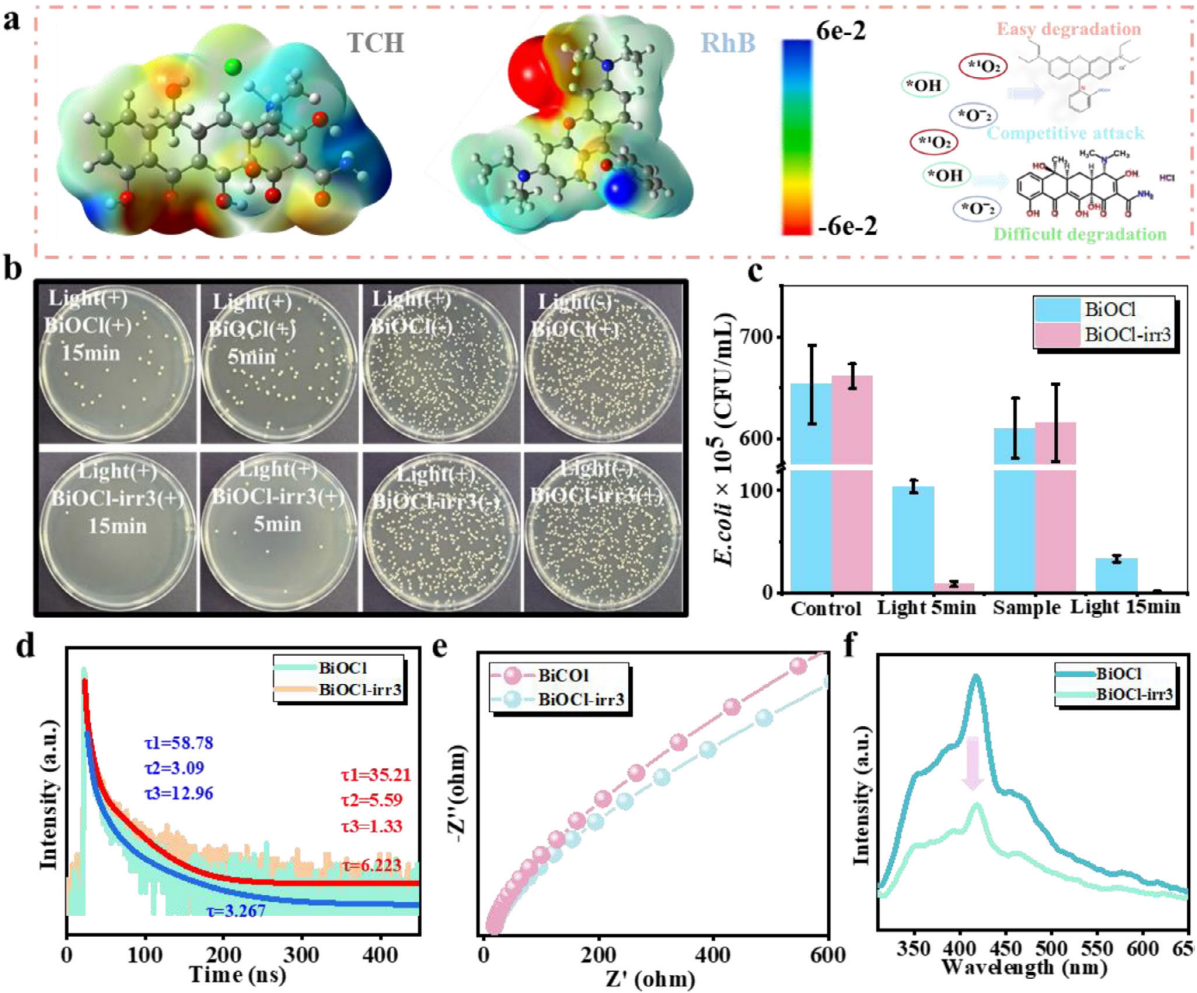
(a) ESP diagram of the TCH and RhB. (b) Photographic images of agar plates. (c) The bacterial viability analysis of *E. coli* incubated with different photocatalysts. (d) The TRPL decay curves of BiOCl and BiOCl‐irr3. (e) Electrochemical impedance analysis of samples. (f) The steady‐state fluorescence spectra of BiOCl and BiOCl‐irr3.

The competitive degradation advantage of RhB over TCH was demonstrated through the surface electrostatic potential (ESP), as shown in Figure 4a. The electrostatic potential is a fundamental physical quantity that delineates the distribution of potential energy within an electric field, directly mirroring the force applied to each unit of positive charge throughout the space. Within an electrostatic field, the electrostatic potential at any given location can be understood as an indicator of the potential energy, representing the work needed to transport a unit positive charge from an infinite distance to that specific point. The negatively charged groups are depicted in red, while the positively charged groups are shown in blue. These negatively charged segments are particularly susceptible to attack by ROS. Consequently, during the photocatalytic degradation process, RhB is more prone to being targeted by reactive oxygen groups, leading to its swift degradation [6, 15, 63]. The theoretical findings align harmoniously with the experimental outcomes.

### 2.3 Photocatalytic Disinfection

The BiOCl‐irr3 catalyst, which has undergone modification via irradiation engineering, exhibits notably satisfactory photocatalytic performance. In order to further unlock and enhance its potential for application in complex and challenging wastewater treatment systems, it is essential to conduct in‐depth research focusing on the integration and utilization of antibacterial systems. The varying degrees of hydrophilicity and hydrophobicity exhibited by surfaces exert a profound influence on bacterial adsorption and the overall antibacterial efficacy [64]. As illustrated in Figure S25, the material progressively develops hydrophobic characteristics following irradiation. This transformation can be attributed to the persistent bombardment of high‐energy particle beams upon the catalyst's surface, which leads to the degradation of hydrophilic groups and subsequent modifications to the surface architecture. As shown in Figure 4b, an analysis of bacterial growth using agar plate photographs reveals that BiOCl‐irr3 exhibits remarkable antibacterial capabilities. With an extension in the duration of light exposure, it becomes evident that nearly all bacteria have been effectively eradicated. A precise bacterial enumeration was carried out, with the outcomes presented in Figure 4c. Following a 15 min duration of light exposure, it was evident that *E. coli* had been nearly entirely eradicated. Notably, only the experimental groups subjected to light exposure alone or those co‐incubated with the BiOCl‐irr3 catalyst failed to exhibit significant antibacterial effects. Furthermore, when compared to BiOCl, the BiOCl‐irr3 catalyst demonstrated a notably superior antibacterial performance. Through the utilization of confocal fluorescence microscopy images, a detailed examination of bacterial viability under various treatment conditions was undertaken. As depicted in Figures S26 and S27, the presence of red fluorescence signifies bacterial mortality, whereas green fluorescence denotes bacterial survival. The findings reveal that following a 15 min treatment involving BiOCl‐irr3 under light exposure, *E. coli* was entirely inactivated. The assessment of relative long‐term bacterial inhibitory capacity was thoroughly considered. Figures S28 and S29 present the bacterial growth vitality curves for various treatment groups. Notably, when both BiOCl and BiOCl‐irr3 were subjected to only a 5‐min irradiation period, the bacteria exhibited rapid growth in the short term. However, the long‐term antibacterial efficacy of BiOCl proved to be even less effective. In contrast, when the light exposure duration was extended to 15 min, the experimental group treated with BiOCl‐irr3 demonstrated no sustained bacterial proliferation within the designated timeframe, thereby underscoring its dependable long‐term antibacterial performance. The morphological characteristics of the bacteria were meticulously captured using SEM, enabling us to observe notable structural alterations. The SEM image presented in Figure S30 provides further corroboration of the aforementioned antibacterial findings. Specifically, the integrity of the bacterial morphology was significantly compromised under illumination following treatment with BiOCl‐irr3. These results compellingly suggest that membrane damage induced by photocatalytic activity constitutes a pivotal factor in achieving highly effective bactericidal outcomes. Furthermore, the membrane damage incurred allows ROS to penetrate into the cellular interior, where they interact with vital genetic and reproductive components such as DNA and proteins. This interaction results in DNA fragmentation and the inactivation of crucial proteinaceous substances, thereby intensifying the programmed demise of the bacteria [23, 65, 66, 67].

### 2.4 The Enhanced Photocatalytic Mechanism of BiOCl Modified by Copper Ion Irradiation

**FIGURE 5:**
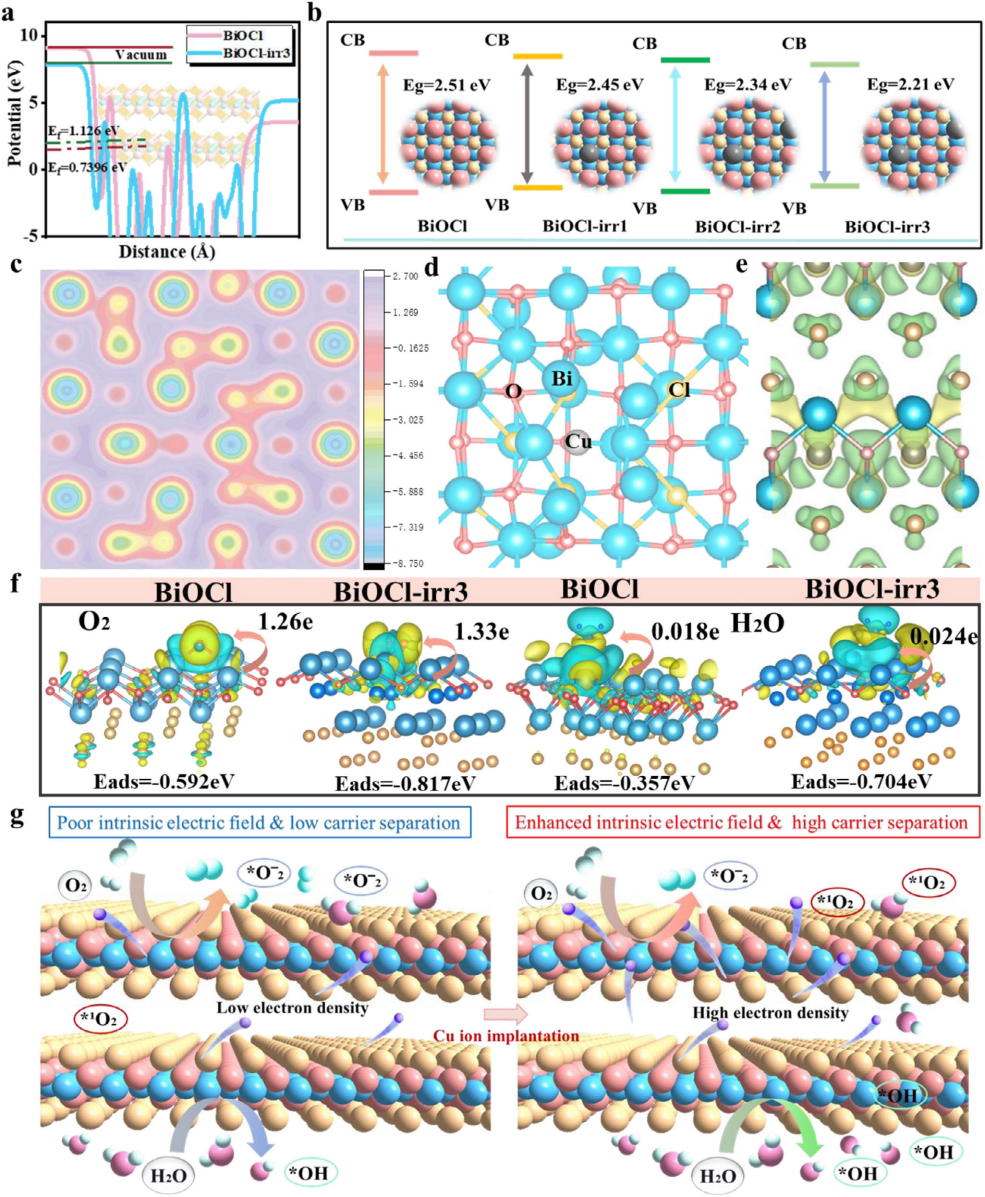
(a) Work function calculated by DFT. (b) Schematic illustration of energy band gaps induced by Cu ion implantation. (c) Average surface electrostatic potential distribution of BiOCl‐irr3. (d) Schematic diagram of the surface electrostatic potential modeling model. (e) Charge density different in BiOCl‐irr3 (Green represents the accumulation of charges, yellow represents the consumption of charges). (f) O_2_ and H_2_O adsorption energy, charge differential density distribution, and the electron transfer quantity (ETQ) on BiOCl and BiOCl‐irr3 (Yellow represents the accumulation of charges, blue represents the consumption of charges). (g) Schematic illustration of enhanced catalytic activity.

To deepen our understanding of the enhanced photocatalytic mechanism of BiOCl‐irr3 and to offer robust methodological guidance for enhancing photocatalytic performance, we carried out a range of photovoltaic performance tests and theoretical analyses on various samples. As seen in Figure 4d, the results of transient fluorescence lifetime measurements reveal that the carrier lifetime in BiOCl is notably shorter compared to that in BiOCl‐irr3, suggesting that ion implantation boosts the separation efficiency of photogenerated electrons and holes, thereby extending their lifetimes. This enhancement in carrier dynamics is intrinsically linked to the substantial improvement observed in the photocatalytic degradation activity. Figure 4e displays the outcomes of the EIS impedance measurements. Notably, the BiOCl‐irr3 catalyst exhibits the smaller EIS radius, signifying that the modification through ion implantation engineering has effectively diminished the electrochemical impedance of BiOCl and facilitated the swift transfer of photogenerated electrons. Based on the carrier recombination photoluminescence theory, a diminished intensity of the photoluminescence peak signals a reduced quantity of recombined electron‐hole pairs. Evident from Figure 4f, BiOCl‐irr3 exhibits a notably weaker fluorescence signal in contrast to the BiOCl catalyst. This observation implies that BiOCl‐irr3 demonstrates superior efficiency in segregating photogenerated carriers, thereby showcasing enhanced photocatalytic performance. Electrons will transfer at the interface between different work functions to attain a new electronic equilibrium state. Figure 5a illustrates the computed work functions of BiOCl and BiOCl‐irr3. Notably, the work function of BiOCl‐irr3 is lower than that of the original BiOCl, signifying that charge transfer has occurred at the interface, thereby enhancing the surface potential. This enhancement is advantageous for further strengthening the intrinsic electric field. This robust theoretical outcome exhibits a pronounced alignment with the findings derived from Kelvin probe force microscopy measurements (Figure 2e). Ion implantation incorporates impurities that modify the energy band structure of BiOCl (Figure 5b, Figures S31 and S32), subsequently impacting its light absorption response range and ultimately boosting the efficiency of light utilization. When juxtaposed with pristine BiOCl, the ion‐implanted BiOCl exhibits a notable shift across the Fermi level, thereby augmenting its conductivity and fostering the swift migration of electrons. Concurrently, the electronic architecture of BiOCl undergoes a profound reconfiguration post‐ion implantation, giving rise to the emergence of novel peaks and alterations in peak morphologies. This transformation is accompanied by an increase in the electron density proximate to the Fermi level, a phenomenon that can provide advantageous in optimizing the electronic dynamic environment (Figures S33–S35). Figure 5c presents an analysis of the electrostatic potential among various atoms, revealing that the Bi atom exhibits a comparatively elevated local electrostatic potential, whereas the O and Cu atoms possess a lower local electrostatic potential (Seen in Figure 5d model). Charges naturally migrate from regions of high electrostatic potential (acting as electron donors) to those of low electrostatic potential (functioning as electron acceptors). Consequently, following ion implantation, electrons migrate from the Bi atom to the O and Cu atoms, thereby facilitating the enhanced separation of charge carriers. This outcome aligns with the findings derived from XPS analysis. Figure 5e illustrates the differential charge density distribution within BiOCl‐irr3, revealing a notable depletion of charges at the Bi atoms and a concurrent accumulation at the O and Cu atoms. This observation further underscores the efficacy of electron separation and migration in bolstering catalytic activity because of the intrinsic electric field enhancement function. Furthermore, we developed an optimized geometric model to simulate the adsorption of H_2_O and O_2_ on BiOCl and BiOCl‐irr3 following Cu ion irradiation. Our findings reveal that BiOCl‐irr3 exhibits a notably stronger binding affinity for H_2_O and O_2_ compared to the pristine BiOCl, with corresponding H_2_O and O_2_ adsorption energies (E*_ads_*) measuring −0.357 and −0.592 eV for the pristine BiOCl, with corresponding H_2_O and O_2_ adsorption energies (E*_ads_*) measuring −0.704 and −0.817 eV for the pristine BiOCl‐irr3, respectively (Figure 5f, Figure S36). BiOCl‐irr3, characterized by its lower adsorption energy, is capable of adsorbing a greater quantity of active molecules and more effectively harnessing the migrating electrons during the electron transfer process, thereby generating a higher amount of active substances. This indicates that BiOCl‐irr3 exhibits an enhanced capacity to produce photogenerated carriers, which subsequently interact with the substances adsorbed on its surface, leading to the generation of a greater quantity of ROS. The theoretical analysis aligns seamlessly with the outcomes of the EPR tests (Figure 3g–i). In other words, BiOCl‐irr3 theoretically possesses the potential to demonstrate superior catalytic activity. Bader charge analysis further demonstrates that the electron transfer from BiOCl‐irr3 and BiOCl to H_2_O amounts to 0.024 and 0.018 electrons, respectively (Figure 5f). This suggests that the introduction of Cu atoms during the ion irradiation process may enhance the electron transfer pathway [19], aligning with experimental observations. Consequently, differential charge density analysis indicates that a greater number of valence electrons from Cu atoms in BiOCl‐irr3 are transferred to the oxygen atoms in both H_2_O and O_2_ molecules. The compelling results from density functional theory (DFT) calculations substantiate that Cu ion implantation engineering can adeptly manipulate the surface gradient electric potential, thereby fostering intrinsic field enhancement. This enhancement, in turn, facilitates the improved separation and migration of photogenerated carriers, enabling a greater number of carriers to engage with oxygen and water molecules in the surrounding environment (Figure 5g). This interaction generates a plethora of reactive oxygen free radicals, culminating in exceptional photodegradation and antibacterial capabilities.

## 3 Conclusion

In summary, the catalytic activity of BiOCl was precisely modulated via controlled copper ion implantation, which tailored its energy band structure to optimize photon utilization efficiency. This modification effectively altered the surface potential gradient of BiOCl, substantially enhancing the intrinsic electric field and facilitating the separation and migration of photogenerated charge carriers. Consequently, the production of ROS was significantly elevated, leading to marked improvements in both photodegradation and antibacterial performance. Compared to pristine BiOCl, the quantitatively irradiated BiOCl‐irr3 exhibited a ∼2.5‐fold enhancement in RhB photodegradation activity and a ∼70% improvement in TCH degradation efficiency, demonstrating superior photocatalytic performance. Within 15 min of light exposure, *E. coli* inactivation reached near‐complete levels (>99%), while maintaining sustained long‐term antibacterial efficacy. These findings provide critical guidance for the rational design of photocatalysts with precisely tuned photodynamic activity, enabling advanced applications in environmental remediation.

## 4 Experimental Section

### 4.1 Synthesis of Pristine BiOCl

BiOCl nanosheets were synthesized using a straightforward one‐step solvothermal approach. Initially, 0.3 g of polyvinylpyrrolidone (PVP) and 0.2 mmol of Bi(NO_3_)_3_•5H_2_O were dispersed in 40 mL of ethylene glycol (EG) solution and thoroughly mixed. Next, 40 mL of a 2 mmol NaCl solution was gradually added to the bismuth nitrate mixture, followed by continued stirring to ensure homogeneity. The resulting mixture was then transferred to a 100 mL reaction vessel and heated at 160°C for 4 h. Once the reaction system had cooled naturally to room temperature, the solution underwent centrifugal purification and was subsequently dried, yielding BiOCl powder.

### 4.2 Cu Ion Implantation

Begin by dispersing the BiOCl powder uniformly in ethanol. Subsequently, accurately obtain 2 mg of the BiOCl and apply it evenly across a 3 cm × 3 cm conductive glass substrate. Allow the liquid to evaporate completely, resulting in the formation of a thin film. Once dried, transfer the film into the ion beam chamber for irradiation. Prior to the irradiation process, it is essential to conduct SRIM simulations to meticulously determine the parameters for the experimental setup (Tables S1–S3). For this study, copper ions with an energy level of 400 keV were selected. Depending on the varying irradiation doses administered (dose: 5 × 10^12^, 1 × 10^13^, and 5 × 10^13^ ions cm^−2^), the post‐irradiation samples were designated as BiOCl‐irr1, BiOCl‐irr2, and BiOCl‐irr3, respectively.

### 4.3 Characterization and Analysis

The morphological features of the material were comprehensively examined using scanning electron microscopy (SEM, HITACHI SU8010), transmission electron microscopy (TEM, TECNAI G20 F30 TWIN, Oxford), and atomic force microscopy (AFM). Its crystallographic structure was elucidated through analyses conducted with an X‐ray diffraction analyzer (XRD) and X‐ray photoelectron spectroscopy (XPS). The photoelectrochemical properties were evaluated utilizing an electrochemical workstation alongside ultraviolet‐visible absorption spectroscopy (UV‐vis abs). Furthermore, the identification and analysis of reactive oxygen free radicals were carried out using an electron spin resonance spectrometer (ESR).

### 4.4 Degradation of RhB and TCH

The solutions of Rhodamine B (RhB) and Tetracycline hydrochloride (TCH) were prepared at two distinct concentrations: 10 and 20 mg L^−1^. Subsequently, the pristine BiOCl and the irradiated BiOCl‐irr3 samples were separately immersed in these pollutant solutions. Following a 60‐min dark reaction period without light exposure, the various degradation systems were subjected to illumination using a 75 W LED lamp. Samples were collected at 20‐min intervals to measure the absorbance of the solutions. To explore different light intensities, the light power was adjusted using a 50 W LED lamp and a 300 W xenon lamp, with the same experimental procedure repeated to obtain absorbance readings for the different pollutant concentrations. Additionally, natural light degradation was investigated by combining RhB and TCH solutions and placing them outdoors for photocatalytic treatment. At various time intervals, treated samples were withdrawn for absorbance analysis. The photocatalytic degradation efficiency was determined using the following formula:

$$\mathrm{Degradation}\;\mathrm{efficiency}\;\left(\%\right)=\left(1-\frac{C_{t}}{C_{0}}\right)\times 100\%$$

In the degradation efficiency calculation, C_0_ corresponds to the initial absorbance of the pollutant solution, while C_t_ signifies the absorbance recorded at subsequent time points during or after irradiation.

### 4.5 Bactericidal Ability

10^8^ colony‐forming units (CFU) of bacteria were extracted and uniformly dispersed onto a conductive glass slide pre‐coated with the sample membrane. The slide was then exposed to 75 W LED irradiation for two distinct durations: 5 min and 15 min. Following irradiation, the illuminated surface film was transferred to a sterile culture medium, thoroughly mixed, and diluted 1000‐fold. Subsequently, 100 µL of the diluted bacterial suspension was plated onto LB agar and incubated at 37°C for 12 h. The resulting colonies were enumerated and photographed for qualitative analysis. Additionally, the diluted bacterial solution was inoculated into liquid LB medium, and bacterial growth was monitored over time by measuring optical density at 600 nm (OD_600_).

### 4.6 DFT Calculation

All calculations were performed using the Vienna Ab‐initio Simulation Package (VASP), which operates on first‐principles calculations grounded in density functional theory. To accurately describe electron exchange‐correlation, the generalized gradient approximation (GGA) was employed, incorporating the Perdew‐Burke‐Ernzerhof (PBE) exchange‐correlation potential. The electron wave functions were expanded using a plane wave basis set with a cutoff energy of 520 eV. Additionally, Grimme's DFT‐D3 method was applied to correct for van der Waals interactions between layers. For k‐point sampling in the first Brillouin zone, a Γ‐centered Monkhorst‐Pack scheme with 6 × 6 × 3 meshes was utilized. The model with a vacuum layer larger than 15 Å was adopted to calculate electrostatic potential, and the dipole correction was applied. The convergence criterion for structural optimization is that the force on each atom in the model is less than 0.02 eV Å^−1^, and the total energy was converged to 10^−5^ eV.

## Conflicts of Interest

The authors declare no conflicts of interest.

## Supporting information




**Supporting file**: advs74050‐sup‐0001‐SuppMat.docx

## Data Availability

The data that support the findings of this study are available from the corresponding author upon reasonable request.
