# Supplementary material for: Enhanced Photocatalytic Wastewater Purification and Bacterial Elimination via Copper Ion Irradiation‐Induced Modification of BiOCl
Source: Adv Sci (Weinh). 2026 Jan 22;13(19):e23633. doi: 10.1002/advs.202523633 (PMC13045436; doi:10.1002/advs.202523633)
Supplement: Supplementary file 1 — Supporting file: advs74050‐sup‐0001‐SuppMat.docx [file ADVS-13-e23633-s001.docx]

# Enhanced Photocatalytic Wastewater Purification and Bacterial Elimination via Copper Ion Irradiation-Induced Modification of BiOCl

Sihan Ma^a,*^, Dewang Cui^b^, Jianglong Kong^c^, Wentao Li^a^, Zheng Han^d^, Yipeng Li^b,e^, Shidong Zhang^a^, Xinglin Yu^a^, Deng Long^a^, Xue Bai^f^, Lin Wang^g,*^, Guang Ran^b,*^ Zhijun Zhao^h,i,*^

a. College of Big Data and Information Engineering, Guizhou University, Guiyang 550025, China

b. College of Energy, Xiamen University, Xiamen 316002, China

c. Department of Food Nutrition and Safety/National R&D Center Herbal Medicine

Processing, College of Engineering, China Pharmaceutical University, Nanjing 211198, China

d. Qujing University of Medicine & Health Sciences, Qujing 655100, China

e. School of Physics, Beihang University, Beijing 100191, China

f. School of Biomedical Engineering, Capital Medical University, Beijing 100069, China

g. Department of Oncology, Zhongshan Hospital of Xiamen University, School of Medicine, Xiamen University, Xiamen 361004, China

h. Central Laboratory, Peking University First Hospital Ningxia Women and Children’s Hospital (Ningxia Hui Autonomous Region Maternal and Child Health Hospital), Yinchuan 750004, China

i. Third Clinical Medical College, Ningxia Medical University, Yinchuan 750001, China

Corresponding authors:

Sihan Ma: [shma@gzu.edu.cn](mailto:shma@gzu.edu.cn)

Lin Wang: [wanglin_linda82@163.com](mailto:wanglin_linda82@163.com)

Guang Ran: gran@xmu.edu.cn

Zhijun Zhao: z15815z@163.com

**Supporting information**

**
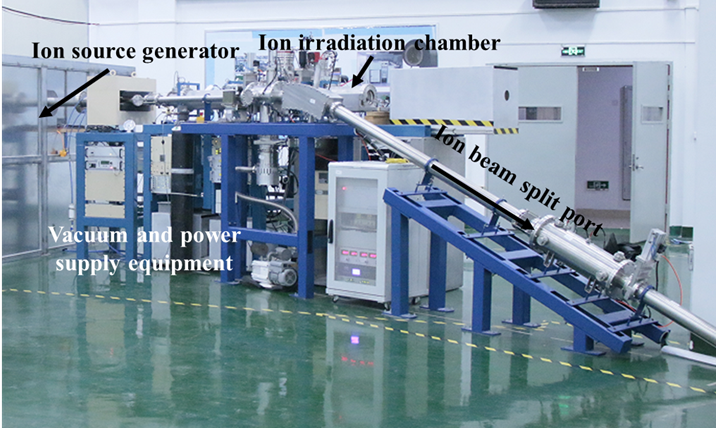
**

**Figure S1.** Ion accelerator facility.


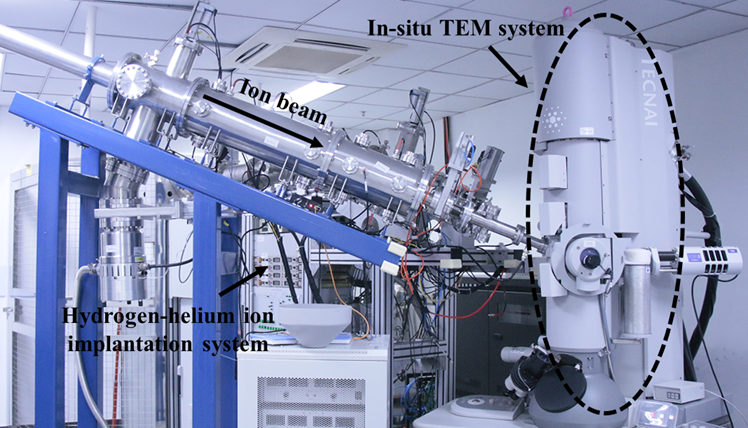


**Figure S2.** Multi-ion beam - in-situ electron microscope linkage facility.


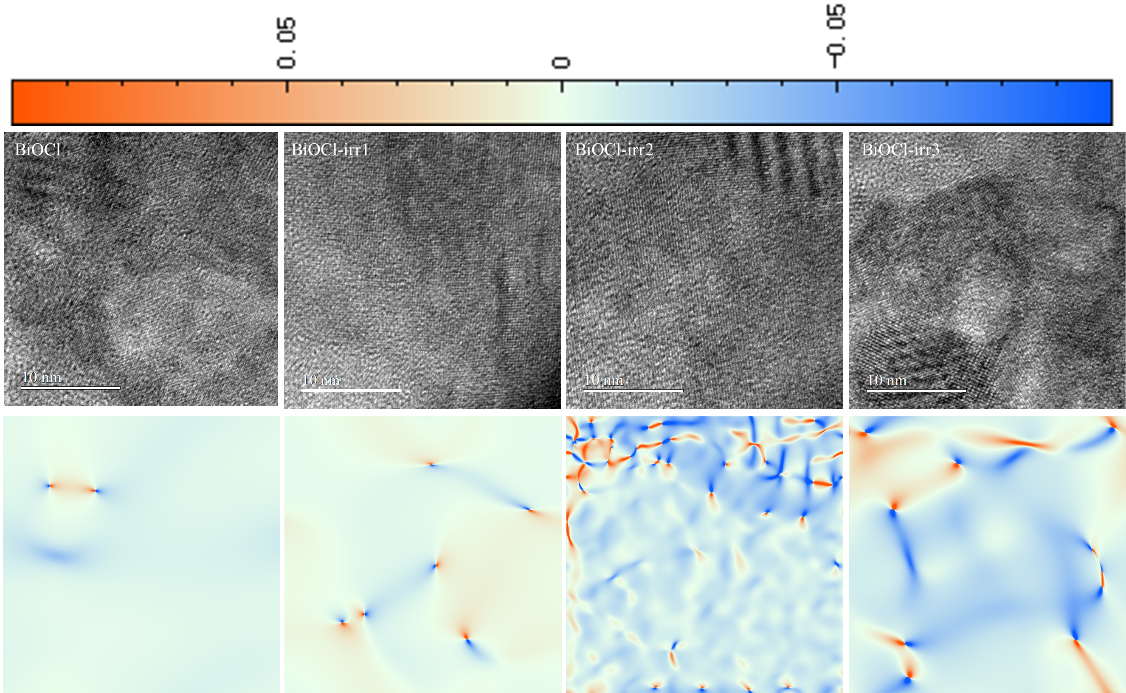


**Figure S3.** HRTEM and ε_xy_ strain images of various BiOCl samples.


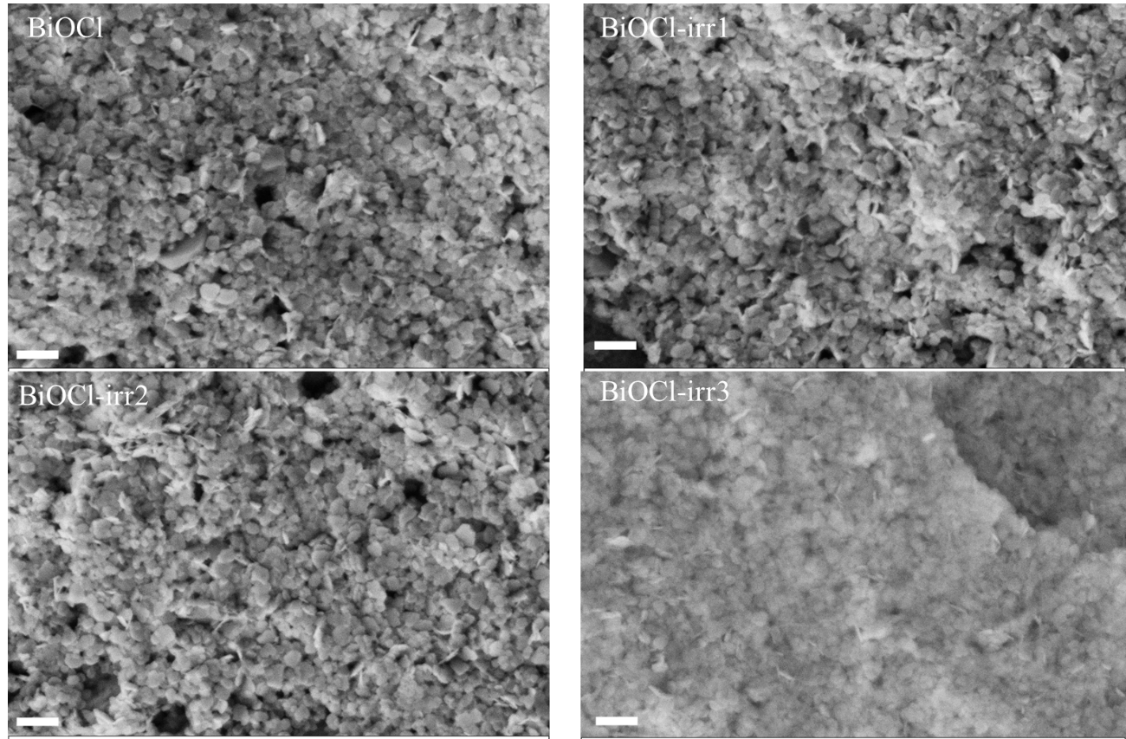


**Figure S4.** SEM images of BiOCl treated with different ion irradiation doses. Scale bar: 200 nm.

**Figure S5.** UV-vis DRS of BiOCl, BiOCl-irr1, BiOCl-irr2 and BiOCl-irr3.


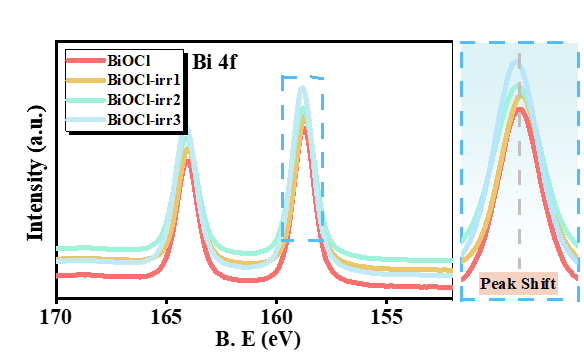


**Figure S6.** XPS refine spectra of BiOCl treated with different ion irradiation doses.


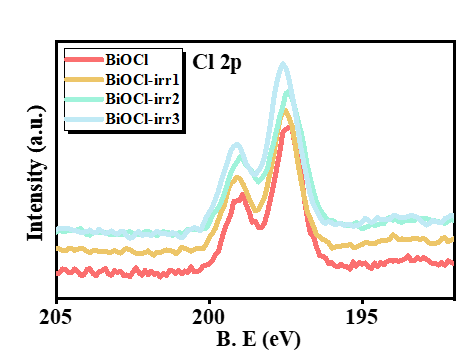


**Figure S7.** XPS refine spectra of Cl *2p* in BiOCl.


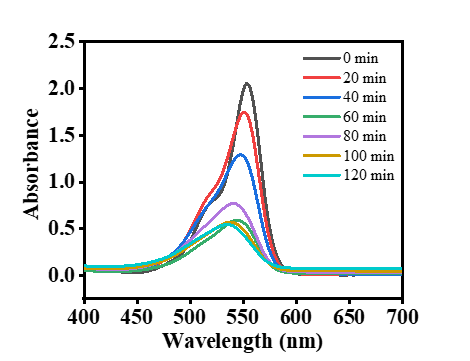


**Figure S8.** Changes in UV-vis spectra during the photocatalytic degradation of RhB solution.


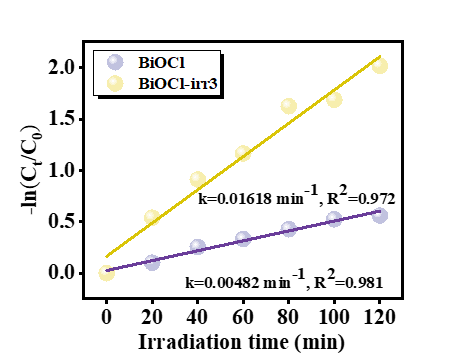


**Figure S9.** Photocatalytic degradation kinetic constant of RhB solution.


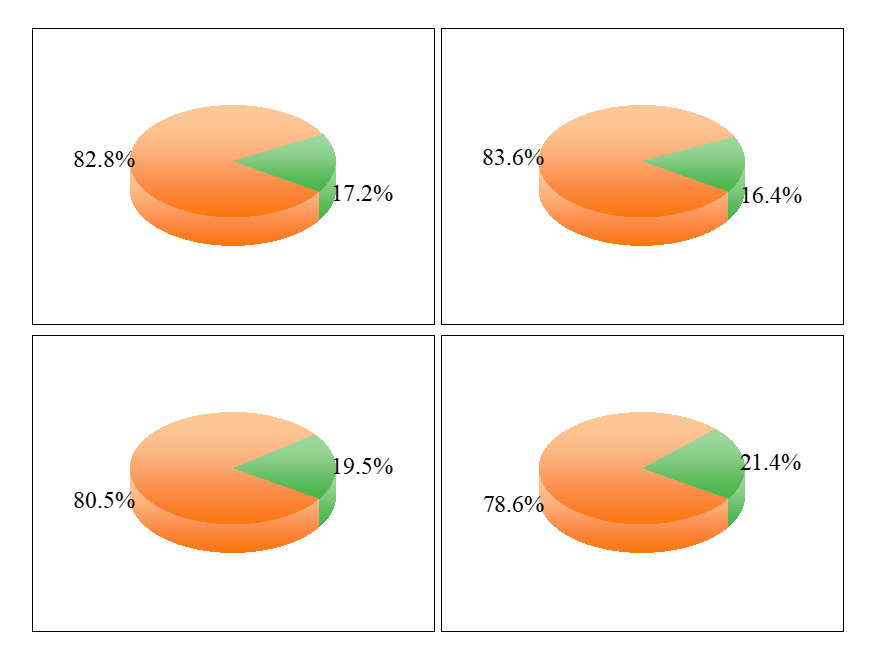


**Figure S10.** Photocatalytic degradation efficiency of RhB solution after four cycles.

**Figure S11.** Photocatalytic degradation curves of RhB solution in tap and pure water.

**Figure S12.** Photocatalytic degradation kinetic constant of RhB solution in various water solution.

**Figure S13.** Photocatalytic degradation kinetic constant of RhB solution when irradiated with various light powers.

**Figure S14.** Analysis of the kinetic constants of the reported RhB catalytic degradation reaction.

**Figure S15.** Changes in UV-vis spectra during the photocatalytic degradation of TCH solution.

**Figure S16.** Photocatalytic degradation kinetic constant of TCH solution.


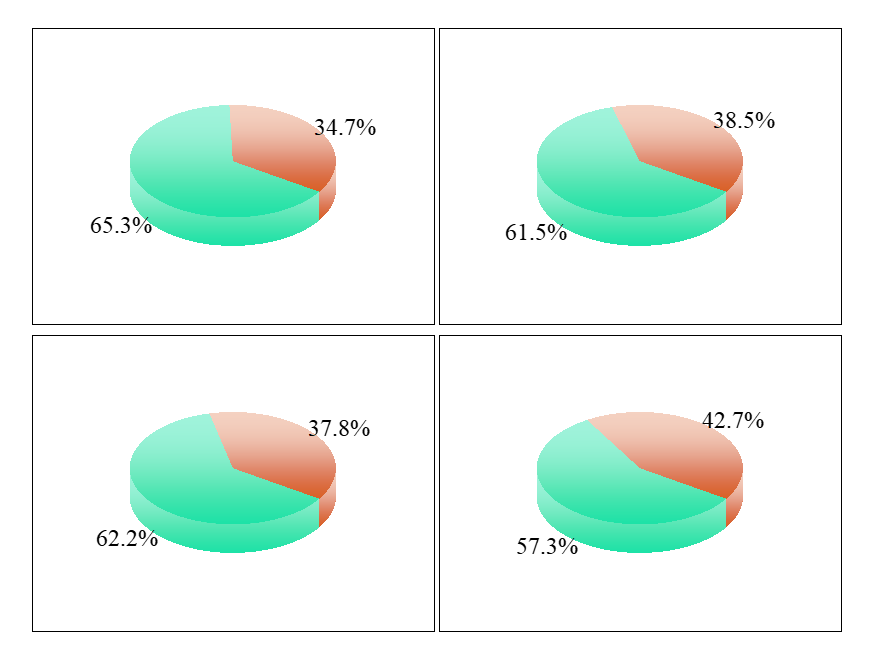


**Figure S17.** Photocatalytic degradation efficiency of TCH solution after four cycles.

**Figure S18.** Photocatalytic degradation curves of TCH solution in tap and pure water.

**Figure S19.** Photocatalytic degradation kinetic constant of TCH solution in different water environment.

**Figure S20.** Photocatalytic degradation curves of TCH solution after different light powers irradiation.

**Figure S21.** Photocatalytic degradation kinetic constant of TCH solution in different light powers.

**Figure S22.** Analysis of the kinetic constants of the reported TCH catalytic degradation reaction.

**Figure S23.** ESR/EPR detection of holes in BiOCl.

**Figure S24.** Detection of holes in BiOCl-irr3.


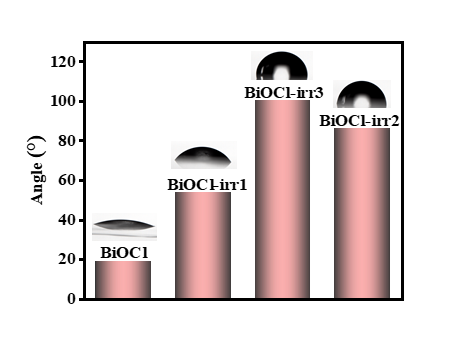


**Figure S25.** Water contact angle of different samples.


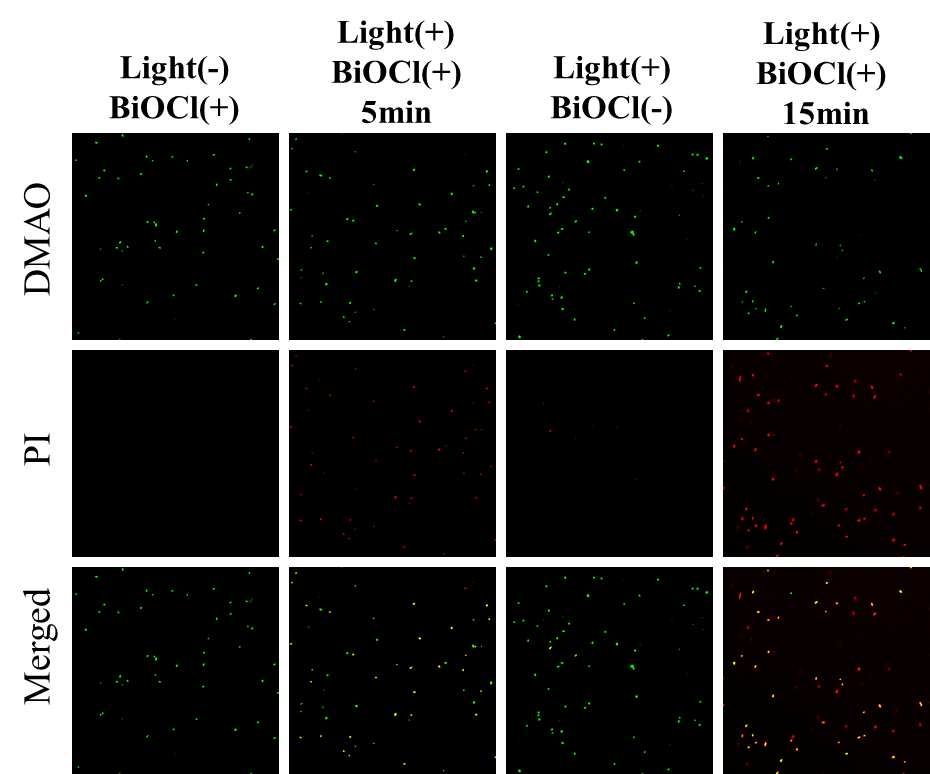


**Figure S26.** CLSM images of *E. coli* incubated with BiOCl.


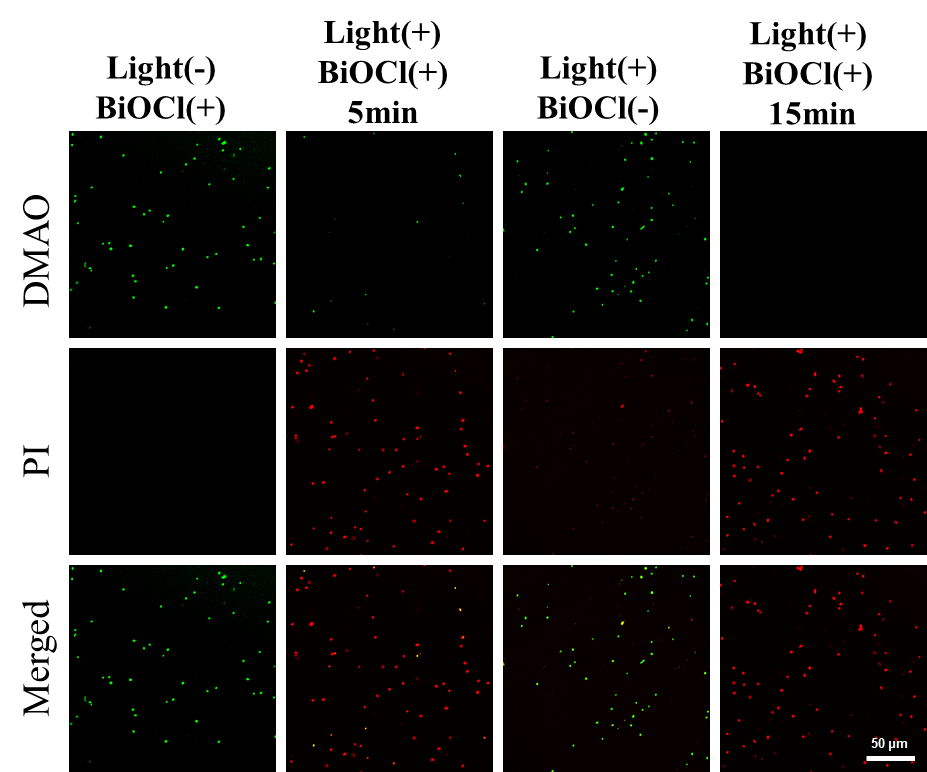


**Figure S27.** CLSM images of *E. coli* incubated with BiOCl-irr3.

**Figure S28.** Antibacterial time-dependent dynamic responding curves of BiOCl against *E. coli*.

**Figure S29.** Antibacterial time-dependent dynamic responding curves of BiOCl-irrs against *E. coli*.


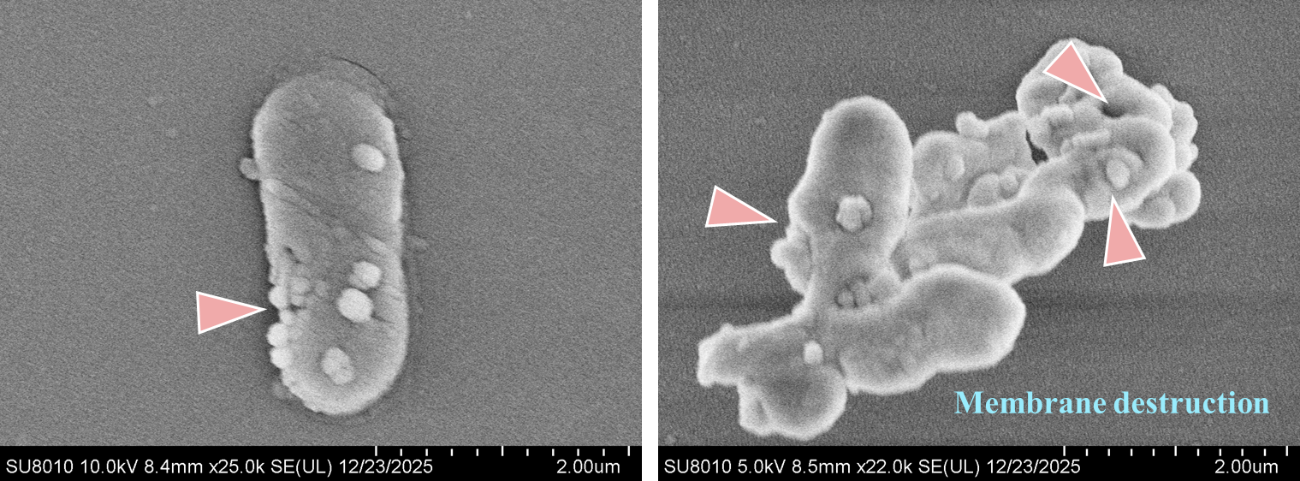


**Figure S30.** SEM images of *E. coli* incubated with BiOCl-irr3 under illumination.

**Figure S31.** DFT calculation of BiOCl energy band gap.

**Figure S32.** DFT calculation of BiOCl-irr3 energy band gap.

**Figure S33.** PDOS of BiOCl.


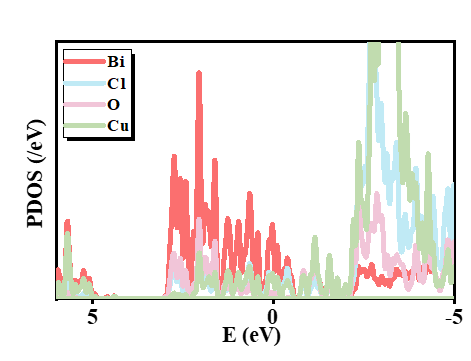


**Figure S34.** PDOS of BiOCl-irr3.


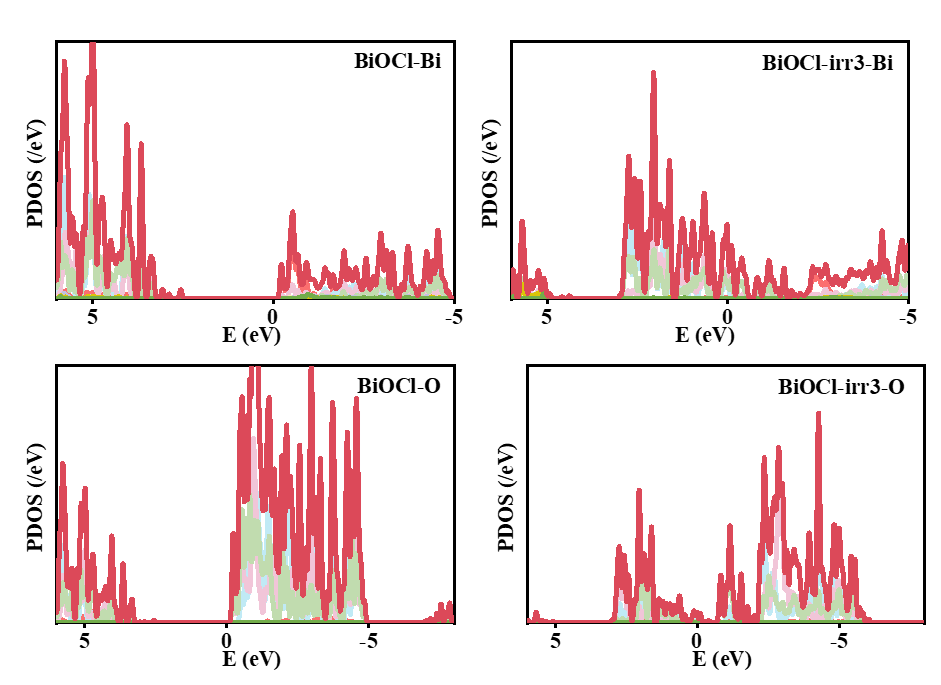


**Figure S35.** PDOS of BiOCl and BiOCl-irr3 for Bi and O.


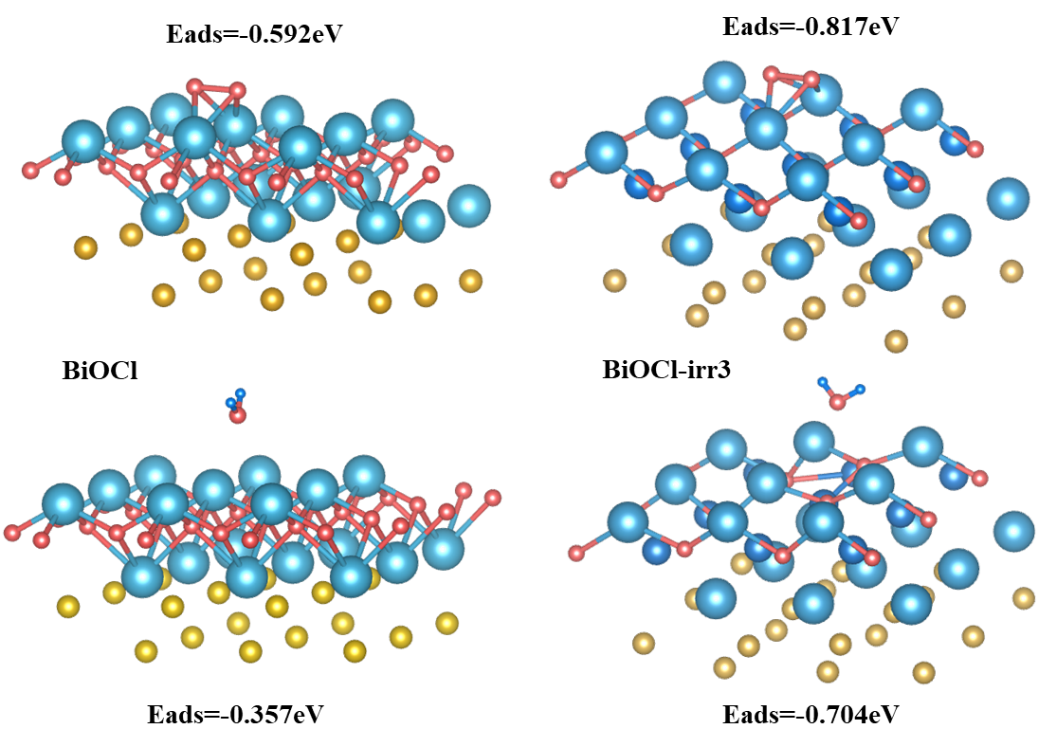


**Figure S36.** O_2_ and H_2_O adsorption energies plots of adsorbed O_2_ and H_2_O in BiOCl and BiOCl-irr3 model.

**Table S1.** Ion implantation plan parameters.

| Sample | BiOCl-irr1 | BiOCl-irr2 | BiOCl-irr3 |
| --- | --- | --- | --- |
| Irradiation dose (ions/cm^2^) | 5×10^12^ | 1×10^13^ | 5×10^13^ |

**Table S2.** SRIM simulation parameters of Cu ions.

| Ion | Energy | Atomic number | Mass(amu) | Angle of incidence |
| --- | --- | --- | --- | --- |
| Cu | 200 keV | 29 | 62.93 | 0 |
| Cu | 400 keV | 29 | 62.93 | 0 |

**Table S3.** SRIM simulation parameters of BiOCl layers.

| Element | Atomic number | Weight | Atomic stoich | Displace | Lattice | Surf |
| --- | --- | --- | --- | --- | --- | --- |
| Bi | 83 | 208.9 | 1 | 23 eV | 3 | 2.17 |
| O  Cl | 8  17 | 15.99  35.45 | 1  1 | 28 eV  25 eV | 3  3 | 2  2 |

**References:**

1. Bui, V. D.; Tran, T. P. A.; Vu, T. H.; Dao, T. P.; Aminabhavi, T. M.; Vasseghian, Y.; Joo, S.-W., Integrating 3D-printed Mo2CTx-UiO-66@rGQDs nanocatalysts with semiconducting BiVO4 to improve interfacial charge transfer and photocatalytic degradation of atrazine. *Applied Catalysis B: Environment and Energy* **2025,** *365*, 124924.

2. Yi, H.; Ma, D.; Huo, X.; Li, L.; Zhang, M.; Zhou, X.; Xu, F.; Yan, H.; Zeng, G.; Lai, C., Facile introduction of coordinative Fe into oxygen-enriched graphite carbon nitride for efficient photo-Fenton degradation of tetracycline. *Journal of Colloid and Interface Science* **2024,** *660*, 692-702.

3. Lu, H.; Zou, F.; Liu, X.; Zhang, W.; Zhang, L.; Deng, C.; Yu, Z.; Monfort, O.; Cheng, P., Z-scheme g-C3N4/α-FOD heterojunction-assisted persulfate activation for degradation of tetracycline hydrochloride under visible light: Insights into mechanism. *Chemical Engineering Journal* **2024,** *479*, 147224.

4. Li, S.; Yang, Y.; Niu, J.; Zheng, H.; Zhang, W.; Leong, Y. K.; Chang, J.-S.; Lai, B., Activation of PAA at the Fe–Nx Sites by Boron Nitride Quantum Dots Enhanced Charge Transfer Generates High-Valent Metal-Oxo Species for Antibiotics Degradation. *Environmental Science & Technology* **2024,** *58* (49), 21871-21881.

5. Jing Li; Dandan Wang; Siyuan Zhao; Rui Ma a, J. G.; Zhuoya Li; Dong Wang; Yue Xuan; Wang, L., Enhanced peroxymonosulfate activation by S-scheme AgI/Cu-BiVO4 heterojunction for efficient photocatalytic organics degradation and Microcystis aeruginosa inactivation: Performance, interfacial engineering and mechanism insight. *Applied Catalysis B: Environment and Energy* **2024,** *351*, 124007.

6. James, A.; Shivakumar; Rodney, J. D.; Joshi, S.; Dalimba, U.; Kim, B. C.; Udayashankar, N. K., Mechanistic insights and DFT analysis of bimetal doped styrofoam-like LaFeO3 perovskites with in-built dual redox couples for enhanced Photo-Fenton degradation of Tetracycline. *Chemical Engineering Journal* **2024,** *481*.

7. Khliwi, F. S.; Alshamsi, H. A., Design of a Z-Scheme System with g-C3N4/WO3/ZnFe2O4 Nanocomposite for Photocatalytic Degradation of Rhodamine B. *J Clust Sci* **2025,** *36* (3), 99.

8. Sun, J.; Liu, H. W.; Wang, S.; Zhang, Y. J.; Bie, C. B.; Zhang, L. Y., irradiated XPS investigation on S-scheme ZnInS@COF-5 photocatalyst for enhanced photocatalytic degradation of RhB. *J Materiomics* **2025,** *11* (3), 100975.

9. Herrera-Mares, M. L.; Jiménez-López, B. A.; Leyva-Ramos, R.; Jacobo-Azuara, A.; Rodríguez-Hernández, J.; Peralta-Rodriguez, R. D.; Galindo-Esquivel, I. R.; Mendoza-Mendoza, E., Novel p-ZnCo2O4/n-Bi2WO6 heterojunctions for efficient rhodamine B and tetracycline photodegradation, and Cr(VI) photoreduction under visible LED and sunlight irradiation. *Ceramics International* **2025,** *51* (7), 9209-9223.

10. Yang, H.; Han, L.; Hou, D. R.; Liu, Y. C.; Huang, S. J.; Peng, Z. R.; Yang, Z. R.; Lu, T. L.; Si, X. Q.; Liu, X.; Li, J. R.; Wang, J. F., Performance and mechanism study on the degradation of RhB by I-g-C3N4@MoO3 heterojunction. *Applied Surface Science* **2025,** *692*.

11. He, Y.; Lin, W. S.; Gao, R.; Zhang, Y. H.; Lin, H. X., Enhanced photocatalytic degradation of rhodamine B pollutants by Ag-deposited BiOI. *Journal of Materials Research* **2025,** *40* (11), 1731-1743.

12. Dhiman, P.; Sharma, J.; Kumar, A.; Verma, Y.; Sharma, G.; Kondal, N.; Lai, C. W., Fabrication of Bi4O5I2/CuO S-scheme heterojunction photocatalyst with enhanced visible light degradation of Rhodamine B. *Journal of Alloys and Compounds* **2025,** *1014*, 178793.
